# In Memoriam: Professor Iya Kiknadze (1930–2017)

**DOI:** 10.3897/CompCytogen.v12i1.24550

**Published:** 2018-03-23

**Authors:** Paraskeva Michailova, Valentina Kuznetsova, Snejana Grozeva, Julia Ilkova, Ninel Petrova

**Affiliations:** 1 Research Group Cytotaxonomy and Evolution, Institute of Biodiversity and Ecosystem Research, Bulgarian Academy of Sciences, 1 Tsar Osvoboditel blvd, 1000 Sofia, Bulgaria; 2 Department of Karysystematics, Zoological Institute, Russian Academy of Sciences, Universitetskaya emb. 1, 199034 St. Petersburg, Russia

With very deep sympathies and very great regret we announce that our best friend, the dearest colleague and excellent researcher Professor Iya Kiknadze passed away in Novosibirsk on December 17, 2017. She was a scientist who made a great and very valuable contribution in the field of cytology – structure and function of the eukaryotic chromosome, cytogenetics and karyotype evolution of family Chironomidae.

Iya Kiknadze was the founder of a very important field in the Cell Biology in Russia – studies of the functional and structural organization of the salivary gland polytene chromosomes in Diptera, especially in the Chironomidae. In many of her studies she followed gene activity during ontogenesis (Kiknadze 1975, Kiknadze et al. 1989). One of the most important and impressive studies was dedicated to gene activity of the key structures of the polytene chromosomes: Balbiani Ring (BR) and Nucleolus Organizer Region (NOR). Her study on the transcriptional activity of these structures represents a great contribution to the genome research on the Chironomidae. Even more, the study on the functional activity of these structures during larval development of the model Chironomid species *Chironomus riparius* Mg. (Kiknadze 1978) provides valuable information about the processes of the environmental mutagenesis. The chromosome map of this species developed by Iya Kiknadze and her collaborators (Kiknadze et al. 1991) is successfully used in the field of genotoxicology for monitoring the environment and for assessing the potential environmental impact (Michailova et al. 2012). Her own study on the chromosome alterations of Chironomids from Republic of Sakha (Yakutia) showed the sensitivity of their genomes to different stress agents in the environment. Her beneficial monograph “Functional organization of the chromosomes” (Kiknadze 1972) presented very well the gene activity and functional organization of the salivary gland chromosomes of the Chironomidae. Dealing with chironomid polytene chromosomes, Iya Kiknadze was the first who has proved that the evolutionarily conserved structure, NOR, is a transcriptionally active region of the interphase chromosomes. Most significant and marvelous studies of Iya Kiknadze on the functional organization of the genomes were done and published in collaboration with well-known specialists as Drs B. Daneholdt, M. Lezzi, J. Edstrom, and U. Grossbach. Together with other scientists, Drs T. Hankeln, E. Schmidt, and her collaborators Drs M. Filippova and K. Aimanova, Professor Iya Kiknadze discovered significant differences in the amount of centromeric heterochromatin between sibling species of the *Chironomus plumosus* group. By *in situ* hybridization they studied the localization and genomic organization of a Sau3AI restriction site (Sau elements) in 24 *Chironomus* Mg. species (Hankeln et al. 1994).

**Figure:**
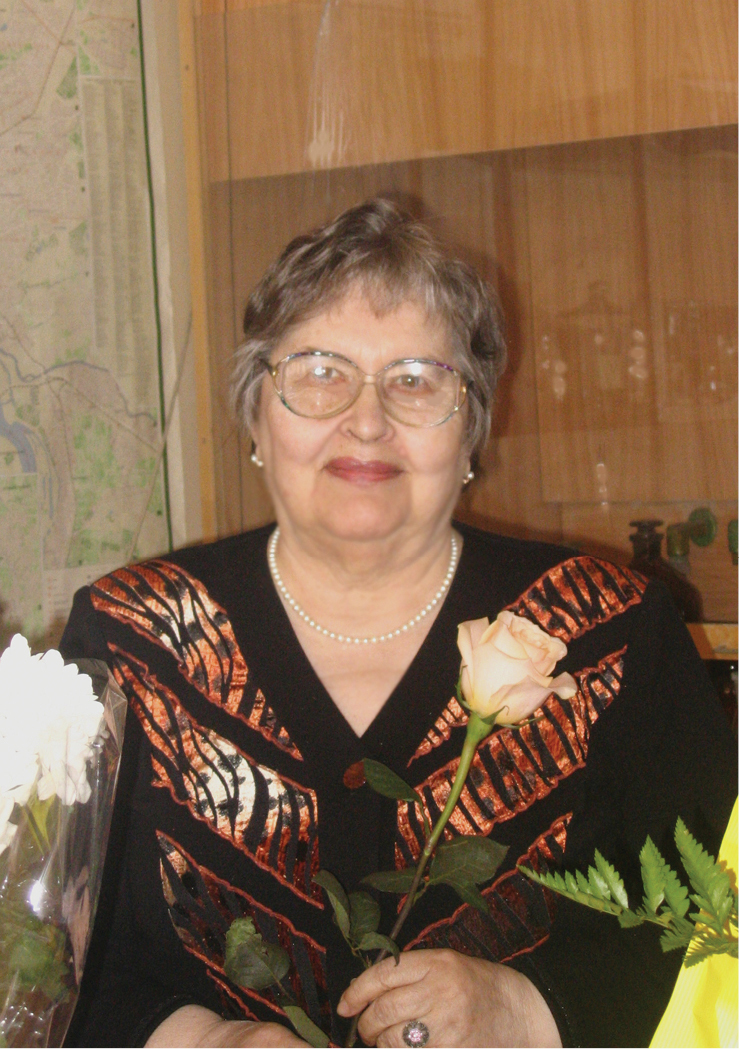
Professor Iya Kiknadze (1930–2017). Photo credit: AG Istomina.

## 

As discussed above, the presence of giant polytene chromosomes in chironomid larvae makes them prospective subjects for genetic, cytogenetic, biochemical and molecular studies. However, to realize the full potential of these subjects precise species identification is necessary. In many cases, a conventional morphological method does not help to identify the species in the larva stage, as the larvae have no distinct differences in external morphology in some genera, e.g. genus *Chironomus* which is in fact rich in sibling species. In all those cases for solving different taxonomic problems of these insects, together with external morphology the markers of the salivary gland chromosomes are being applied. Iya Kiknadze was the author or a co-author of many original chromosome maps of different Chironomid species (Kiknadze et al. 1991, 1996) where banding patterns of polytene chromosomes have been combined with detailed morphological analyses of larvae. These maps allow the scientists to solve many problems in taxonomy, cytogenetics, and chromosomal polymorphism of natural populations of the species, to trace the path of species divergence. Based on the chromosome maps, the rearrangements in the polytene chromosomes and different biomarkers are applied to analyze precisely the genome response to various stress agents in the environment. Of especial interest is her book “Karyotypes of Palearctic and Holarctic species of the genus *Chironomus*” (Kiknadze et al. 2016), where Iya Kiknadze, together with her closest colleagues Drs A. Istomina, V. Golygina and L. Gunderina, presented the cytogenetic characteristics of 63 species of genus *Chironomus*, collected from different geographical areas, including Russia (European part, Ural, West and East Siberia, Altai, Tuva, the Far East), Kazakhstan, West Europe (Germany, Belgium, the Netherlands), Bulgaria, USA, Canada, China, and Japan. The pictures of the polytene chromosomes of all studied species are brilliant and allow very easy and correct species identification as well as analyzing chromosomal polymorphisms, the chromosomal rearrangements involved in the species divergence. Here it is important to underline that phylogenetic analysis of these data allowed her to reconstruct the cytogenetic history of many Chironomid species (Kiknadze et al. 2008). The authors evaluated the role of chromosome aberrations in population divergence and speciation. They found that in each continent there are endemic species-specific sequences together with common sequences for different species and cytocomplexes.

Iya Kiknadze had a very fruitful collaboration not only with colleagues from different parts of Russia (besides some mentioned above, these are Drs S. Belyanina, N. Petrova etc.) but also with specialists from all over the world: Australia – Dr J. Martin, USA – Dr M. Butler, Europe – Germany – Dr W. Wülker, Bulgaria – Dr P. Michailova, Netherlands – Dr H. Vallenduuk etc. As a result, they described together many new chironomid species, presented a number of unique chromosome maps and suggested the main paths of karyotype evolution in the Chironomidae.

Iya Kiknadze was the teacher and supervisor of many entomologists, cytogeneticists, geneticists, karyosystematicists in Russia, especially in Novosibirsk. Her scientific ideas are followed by many scientists in Europe, Asia and America. As a famous scientist and specialist in different fields of Genetics she has been invited in the editorial boards of many journals as Tsitologiya, Ontogenesis, Eurasian Entomological Journal and Comparative Cytogenetics.

Prof. Iya Kiknadze was not only a remarkable scientist but she had a real talent for organization of different Symposia and Conferences. She initiated the first International Symposium on Organization and Expression of Tissue Specific Genes in Novosibirsk in 1982 and it became a regular every second year workshop on Chironomid Balbiani Ring, organized in different countries. An important dimension of her career was the participation in many international Symposia, workshops in the field of structure and function of the genome as well as in the field of Karyosystematics and Evolution of the Chironomidae. Always her presentations were followed with a great interest.

With the death of Professor Iya Kiknadze we lost a great specialist in genetics, cytogenetics and karyosystematics of the Chironomidae. We will miss our best friend and a great person. We will always remember Iya Kiknadze for her work, commitment and energy. She will always live in our hearts and memories.
